# Under Victimization by an Outgroup: Belief in a Just World, National Identification, and Ingroup Blame

**DOI:** 10.3389/fpsyg.2018.01160

**Published:** 2018-07-24

**Authors:** Isabel Correia, Cicero R. Pereira, Jorge Vala

**Affiliations:** ^1^Departamento de Psicologia Social e das Organizações, Instituto Universitário de Lisboa, Lisbon, Portugal; ^2^Departamento de Psicologia, Universidade Federal da Paraíba, Paraíba, Brazil; ^3^Instituto de Ciências Sociais, Universidade de Lisboa, Lisbon, Portugal

**Keywords:** belief in a just world, national identification, victimization, austerity, ingroup blame, financial crisis, recession, identification

## Abstract

Using representative probabilistic samples of Portuguese citizens and framed by an intergroup perspective, we carried out two studies aiming to address how national identification and belief in a just world (BJW) jointly predict secondary victimization of an ingroup as a whole (specifically ingroup blame). We conducted Study 1 (*N* = 779) in 2014, at the height of the European austerity policies imposed on Portugal by an institutional outgroup, specifically the Troika (the European Union, the European Central Bank, and the International Monetary Fund). Study 2 (*N* = 1140) was conducted after the Troika intervention. An environment of ongoing ingroup suffering caused by an outgroup is more threatening for the BJW of individuals who are more identified with the ingroup. We therefore predicted and found that BJW was positively associated with ingroup blame in participants higher in national identification when the victimization provoked by an institutional outgroup was higher (Study 1). However, when the suffering caused by the outgroup decreased, the association between BJW and secondary victimization was not moderated by individuals’ national identification (Study 2). Indeed, a three-way interaction was found between BJW, national identification, and social context (high vs. low victimization). These results are an important contribution for the literature about justice motivation in terms of intergroup relations, because they show that secondary victimization produced by a threat to BJW has a group-based identity function.

## Introduction

In 2011, at the height of the euro crisis, the European Union imposed strong austerity programs on Portugal, as well as on other European countries (Ireland, Greece, Cyprus, Spain, and Italy). In Portugal, specifically, the measures designed by the Troika (the European Union, the European Central Bank, and the International Monetary Fund) have profoundly affected citizens’ lives. The GDP fell significantly between 2010 and 2014, unemployment also significantly increased, and many people emigrated (INE, 2017). In addition to the poverty rate rise, taxes increased, wages decreased, and the investment in health care and education was greatly reduced. The measures imposed by the Troika have brought intense suffering, increased social inequalities, and have been an issue of intense political and economic debate, nationally and internationally (e.g., Greenglass et al., 2014).

How did the Portuguese citizens react to this economic crisis? Did they consider themselves responsible for their social and economic misfortune? Research on the psychosocial variables affecting the reaction of populations during the last global economic crisis has been scarce [Christandl (2013) and Papastamou et al. (2018) are exceptions], especially those associated with ingroup blame for its own suffering. This paper intends to fill this gap by investigating how belief in a just world (BJW, Lerner, 1980) and national identity predict Portuguese citizens’ ingroup blame.

Previous research has shown that when confronted with a victim of injustice, individuals need to preserve their perception that the world is just (BJW, Lerner and Simmons, 1966; Lerner, 1980), so that they can maintain their confidence in the future and guarantee their mental health (Lerner, 1980; Dalbert, 2001). The preservation of the BJW when facing injustice can be done through various ways, such as the engagement in actions perceived as effective in re-establishing justice (Bierhoff et al., 1991), or through “secondary” victimization, i.e., blaming or derogation of the victims, whether the victims are other people (e.g., Sutton and Douglas, 2005, for a review, see Hafer and Bègue, 2005) or the actual individual (e.g., Hafer and Olson, 1989; Choma et al., 2012). This process of cognitive restoration of justice only happens when the suffering is ongoing (Lerner and Simmons, 1966) and it is not possible to alleviate that suffering (Correia and Vala, 2003).

Importantly, recent studies have shown that the more people believe in a just world and the more identified with the victim’s group they are, the higher their need to reestablish the perception of justice when threatened by ingroup victimization. Indeed, those individuals showed higher secondary victimization of an ingroup victim (Correia et al., 2012), or of many members of the ingroup, and possibly they themselves (Correia et al., 2015).

Nevertheless, research has not investigated whether this pattern occurs when individuals themselves are victims simply due to belonging to a given group and when the victimization takes place in an intergroup international context. Based on a national probabilistic sample, this study intends to test whether the BJW and national identification of Portuguese citizens jointly predict the victims’ reactions to the suffering inflicted by the economic measures imposed by the Troika.

## National Identification, Belief in a Just World, and Victimization of Ingroup Members

According to social identity theory (Tajfel and Turner, 1979), when people categorize themselves as members of social groups they define themselves more in terms of their group rather than their personal characteristics. Therefore, sharing a common identity with a victim of injustice is a potential cause of threat to one’s BJW (Novak and Lerner, 1968; Lerner and Miller, 1978; Opotow, 1995; Correia et al., 2007), especially when individuals strongly identify with their own group (Correia et al., 2012). As previously assumed by the social identity approach (Turner et al., 1987), “the degree of internalization of or the identification of a category with an ingroup–outgroup membership […] is a major determinant of accessibility” of a category (Turner et al., 1987, p. 55).

This happens because there is a convergence between the belief that good things happen to good people while bad things happen to bad people (Lerner, 1980), and the motivation for positive distinctiveness arising from the categorization between us, the “good ones”, and them, the “bad ones” (Tajfel and Turner, 1979). A non-threatening situation occurs when bad things happen to the outgroup or when good things happen to the ingroup. On the other hand, a threatening event occurs when bad things occur to good people, i.e., when the ingroup is victimized. In such a situation, individuals are motivated to solve the threatening incongruence by restructuring the situation in order to perceive it as just and legitimate. Furthermore, within the framework of the system justification theory (Jost and Banaji, 1994) research has shown that the need for system justification can lead individuals of disadvantaged groups to blame themselves and their group for their own disadvantage. Although apparently paradoxical, the costs of internalization of inequality at a personal and group level are compensated by the benefits at the system level that one’s outcomes are predictable and controllable (McCoy et al., 2013), which reduces the threat caused by the ingroup misfortune.

Indeed, after results such as those of Novak and Lerner (1968), where victims who were perceived as being more similar to the observer were subject to greater avoidance, Correia et al. (2007) showed that an innocent ingroup victim is more of a threat to the BJW than an innocent outgroup victim. One other study (Aguiar et al., 2008, Study 2), additionally showed that the ingroup victim was also more secondarily victimized in a non-obtrusive, or implicit derogation measure, than an ingroup non-victim.

However, these previous studies did not differentiate between participants who strongly and weakly endorsed the BJW, nor between participants who strongly and weakly identified with the group. The introduction of these measures allowed for findings on explicit derogation measures. It was found that when the identity of the victim and the victimization situation are not necessarily related (e.g., a university student who was run over by a car), the positive relationships between BJW and victims’ derogation, and between BJW and psychological distancing, were significant for strongly identified participants but not for weakly identified participants (Correia et al., 2012). Furthermore, the same result was found when there is an intrinsic relation between being an ingroup member and being a victim (such as in the case of wife abuse, Correia et al., 2015). In the latter case, the measure of reaction toward victims was the legitimation of wife abuse. Therefore, both when the identity of the victim and the victimization situation were and were not related, there was an association between BJW and secondary victimization for strongly identified participants but not for weakly identified participants. This means that the relationship between BJW and secondary victimization of a specific target perceived as an ingroup member is moderated by the extent to which the perceiver identifies with this ingroup.

These previous studies analyzed the victimization of particular individuals in a group but not the victimization of the group as a whole. The current paper goes further by proposing that victim blaming is a more general phenomenon affecting the entire ingroup when it is under suffering. Specifically, we test whether BJW and identification also interact to predict secondary victimization (i.e., ingroup blame) when the group as a whole is a target of misfortune, in this particular case, provoked by the economic austerity measures imposed by the Troika. We may then predict a two-way interaction between BJW and ingroup identification on ingroup blaming, so that the impact of BJW should be stronger in more identified individuals when the ingroup is victimized.

Because only negative events occurring to the ingroup are threatening to BJW, when the ingroup suffering is lower, more identified individuals will no longer be motivated to blame the ingroup because their ingroup is no longer being victimized. In such a situation, ingroup identification should not moderate the impact of BJW on ingroup blame. Accordingly, it is likely that the interaction between BJW and ingroup identification should occur when the ingroup is a target of a misfortune, but not when the ingroup is no longer victimized. We may then predict a three-way interaction between BJW, ingroup identification, and the victimization context.

If this is the case, this paper represents an important contribution for the literature about justice motivation in terms of intergroup relations. For the first time, secondary victimization produced by a threat to BJW will be shown not to be specific to individuals who suffer life misfortunes, but a wider phenomenon that also has a group-based identity function. According to the BJW theory, this happens because blaming of the ingroup when facing victimization is a protective mechanism for people to continue to believe that they, as ingroup members, are protected from injustices, so that they can continue to delay gratification and to invest in the future, hoping that they will be fairly rewarded.

## Overview of Studies

Using representative probabilistic samples of Portuguese citizens, we carried out two studies aiming to address, how national identification and BJW jointly predict secondary victimization of an ingroup as a whole (specifically ingroup blame) when there is a threat to BJW caused by externally imposed austerity measures, and when this threat is lower because those austerity measures have finished. We conducted Study 1 in 2014, at the height of the Portuguese financial crisis during the intervention by the Troika. Study 2 was conducted 3 years later, when the Troika had already left Portugal.

As we already mentioned, this external intervention was accompanied by a profound deterioration of economic activity, with extremely negative consequences for the lives of the population. Indeed, there was the deep economic recession resulting from the Troika’s financial intervention which produced an environment of continued social and psychological suffering that affected almost the entire Portuguese population. This scenario, at the time of Study 1, provided us with a unique opportunity to analyze secondary victimization in a realistic context. When the measures finished, at the time of Study 2 there was a period of some social enthusiasm due to returning economic growth, allowing us to see the social environment in which the study was carried out as one of less suffering compared to that of Study 1.

Together, these two studies allowed us to test the hypothesis that BJW is positively associated with ingroup blaming for its own suffering and that this association is moderated by national identification and by threat produced by ingroup suffering. Specifically, we predicted that, because an environment of ingroup suffering is more threatening of the BJW of more ingroup identified individuals, BJW would be positively associated with ingroup blame in highly identified Portuguese participants, i.e., individuals’ degree of national identification should moderate the effect of BJW on secondary victimization (Study 1). Additionally, we predicted a different pattern of results when the suffering caused by social environment decreased. When external intervention was concluded and the country’s socio-economic situation improved, ingroup suffering should be less salient for national identified individuals and, so, the association between BJW and secondary victimization should not be moderated by individuals’ national identification (Study 2).

## Study 1

In this study, we aimed to address the articulation between national identity and BJW on ingroup blame for economic suffering during externally imposed austerity measures. More specifically, as regards to ingroup blame, we predicted that only for highly identified Portuguese participants, BJW would be positively associated with ingroup blame because the BJW motivates the reestablishment of justice in the world, when the victim shares a common identity with the perceiver.

Additionally, in order to better test our hypotheses several control variables were introduced in the regression models. These variables (socio-demographics, religiosity, and political ideology) have shown to be correlated with BJW in previous studies [see Correia (2003) for a revision] and, consequently, may contribute to explain the dependent variable ingroup blame. This is even more probable if we take into account that the study is cross-sectional and the sample is representative of a countries’ population.

We also predict that European identification may foster ingroup blame. Therefore, it should be controlled so that a stronger test of our hypothesis that BJW and national identification predict ingroup blame over and above those control variables can be done.

### Materials and Methods

#### Participants and Procedure

A national representative probabilistic sample was used (*N* = 1001). For the present study, we only considered data from participants who were Portuguese citizens (*N* = 974). Of these participants, 779 matched all measurements and therefore constitute the final sample of the present study (ages between 18 and 93 years, *M* = 47.67, *SD* = 17.16; 363 male and 416 female; years of schooling, *M* = 9.46, *SD* = 4.77).

Data were collected between September 2014 and January 2015, as part of the Portuguese module of the International Social Survey Program (ISSP). These data were collected by trained interviewers. The interviews were face to face and carried out in the participant’s home. Participants’ consent was obtained prior to the beginning of the study, in accordance with the Declaration of Helsinki. The protocol and questionnaire were approved by the ISSP General Assembly according to their Ethics Statement, except the item that measured ingroup blame and the items that measured Belief in a Just World, that were part of the Portuguese module of the ISSP.

The data and further information about documentation and data collection can be found at http://www.issp.org, and at https://www.ics.ulisboa.pt/docs/issp/Study_1_ISSP_database _Portugal.sav (for the Portuguese Specific Module).

#### Measures

##### Religiousness

We measured this construct with one item asking people “Without counting special occasions such as weddings, funerals, and baptisms, how frequently do you participate in religious services?” in an eight point scale from 1 “Several times a week” to 8 “Never.” The answers to the items were recoded so that higher scores indicate stronger endorsement of the construct.

##### Subjective social status

We measured this construct with one item asking people “In a general way, some people are at the top of our society and others are at the bottom. This scale represents the top and the bottom. At which point of the scale do you think you are at this time of your life?” in a 10-point scale from 1 “Bottom” to 10 “Top.”

##### Belief in a just world

We measured this construct with three-items taken from the General Belief in a Just World Scale (Dalbert et al., 1987) (“I am confident that justice always prevails over injustice;” “I believe that, by and large, people get what they deserve;” “I am convinced that in the long run people will be compensated for injustices; Cronbach’s α = 0.72), ranging from 1 (totally agree) to 5 (totally disagree). The answers to the items were recoded so that higher scores indicate stronger endorsement of the construct. We computed a global score for this scale by averaging across items.

##### Ingroup identification

We used two items to measure ingroup identification (“How close to or identified do you feel with Portugal?” and “How proud do you feel to be a Portuguese citizen”, *r* = 0.31) in a four-point scales ranging from 1 (very much identified) to 4 (not at all identified). The answers were recoded so that higher scores indicate stronger endorsement of the construct. We computed a global score for this scale by averaging across items.

##### European identification

We used one item to measure identification with Europe (“How close to or identified do you feel with Europe?”) in a four-point scales ranging from 1 (very much identified) to 4 (not at all identified). The answers to the item were recoded so that higher scores indicate stronger endorsement of the construct.

##### Ingroup blaming

We measured this construct with one item “Regarding the impact of the measures imposed by the Troika in Portugal, Portuguese people are partly to blame for the suffering they are going through” with five-point scales ranging from 1 (totally agree) to 5 (totally disagree). The answers were recoded so that higher scores indicate stronger endorsement of the construct. Sixty percent of the participants said they agreed or totally agreed with that sentence; only 26% disagreed or strongly disagreed.

### Results and Discussion

#### Preliminary Analysis

**Table 1** presents descriptive statistics and zero-order correlations between all variables. Some results are important for a better characterization of the context in which the study was carried out, besides serving as baseline for comparisons with Study 2. Both national and European identification were strongly higher than the midpoint of the response scale (*t* = 41.31, *p* < 0.001, *d* = 2.77), and (*t* = 21.49, *p* < 0.001, *d* = 1.44), respectively. Mean scores of BJW were slightly below the midpoint of the scale (*t* = -16.48, *p* < 0.001, *d* = -1.10). In turn, ingroup blaming was substantial, being scored significantly above the midpoint of the scale (*t* = 10.88, *p* < 0.001, *d* = 0.73). Concerning the correlations, BJW and national identification were both positively correlated with ingroup blame. Additionally, ingroup blame was positively and significantly correlated with identification with Europe and years of schooling, and negatively correlated with age.

**Table 1:** Correlations and descriptive statistics in Study 1.

Scale	*M*	*SD*	2	3	4	5	6	7	8	9
(1) Sex	–	–	0.08*	–0.07*	0.23***	–0.06	–0.04	0.02	–0.03	–0.02
(2) Age	47.67	17.16	–	–0.58***	0.32***	–0.15***	–0.27***	0.04	–0.08*	–0.08*
(3) Years of schooling	9.46	4.77		–	–0.27***	0.26***	0.31***	–0.06*	0.01	0.11***
(4) Religiousness	3.49	2.20			–	–0.09**	–0.14***	0.09**	0.00	–0.05
(5) Social status	4.92	1.64				–	0.18***	0.06*	0.12**	0.03
(6) European identification	2.71	0.82					–	0.12**	–0.04	0.17***
(7) National identification	3.31	0.54						–	0.08*	0.11**
(8) Belief in a just world	2.61	0.79							–	0.14***
(9) Ingroup blame	3.47	1.15								–

*For all scales higher values indicating stronger endorsement of the construct. For sex, 0 indicates “male” and 1 “female.” ^∗^*p* < 0.05; ^∗∗^*p* < 0.01; ^∗∗∗^*p* < 0.001.*

#### Main Analysis

We then tested whether national identification moderated the relationship between BJW and ingroup blame (the outcome variable), while controlling for the effects of European identification. As age, years of schooling, social status, and religiousness correlated significantly with the main predictor variables (BJW and national identification), as well as with the criterion variable (ingroup blame), they were also introduced in the regression.

We thus conducted a multiple regression analysis. In a first block, we entered the socio-demographic (age, sex, years of schooling, religiousness, social status) and the control variable (European identification). In a second block, we entered BJW and national identification. In a third block, we entered the product between BJW and social identification. In the current and in the subsequent study, all the predictor variables were centered before analyses (Aiken and West, 1991).

The results are shown in **Table 2**. In the final model, ingroup blame was explained by years of schooling, European identification, national identification, and BJW. All these associations were positive. Furthermore, a significant two-way interaction between BJW and social identification significantly predicted ingroup blame. This significant effect obtained in the third model was over and above the effects of other variables included in the model estimated in Block 1 and Block 2.

**Table 2:** Regression of ingroup-blame on controlling variables, BJW, and national identification, and interaction between BJW and national identification (Studies 1 and 2).

	Study 1		Study 2A		Study 2B	
	*b*	*SE_b_*	*b*	*SE_b_*	*b*	*SE_b_*
Intercept	3.50	0.14	3.08	0.05	2.35	0.04
Controlling variables					
Sex	0.00	0.00	0.15	0.10	–0.13	0.08
Age	0.00	0.08	–0.01	0.01	–0.01	0.01
Years of schooling	0.02^∗^	0.01	0.00	0.01	0.01	0.01
Religiousness	–0.01	0.02	–0.01	0.03	0.02	0.02
Subjective social status	–0.03	0.03	0.07	0.06	0.00	0.05
European identification	0.19^∗∗∗^	0.05	0.06^∗^	0.03	0.04	0.02
Left–right			0.05^∗^	0.02	0.03	0.02
$R_{\mathrm{Adjusted}}^{2}$	0.03^∗∗∗^		0.03^∗∗^		0.03^∗∗∗^	
Theoretical predictors					
National identification	0.21^∗∗^	0.08	–0.05	0.10	–0.01	0.08
Belief in a just world	0.19^∗∗∗^	0.05	0.30^∗∗∗^	0.07	0.13^∗^	0.06
$R_{\mathrm{change}}^{2}$	0.03^∗∗^		0.04^∗∗∗^		0.01^∗^	
Interaction term					
BJW × national identification	0.24^∗∗^	0.10	0.01	0.12	–0.09	0.11
$R_{\mathrm{change}}^{2}$	0.01^∗∗∗^		0.00		0.00	
$R_{\mathrm{Adjusted}}^{2}$	0.07^∗∗∗^		0.06^∗∗∗^		0.03^∗∗∗^	

*b, unstandardized coefficients. For all measures, scores were computed by averaging across items, with higher scores indicating stronger endorsement of the construct. For sex: 0 = “male”; 1 = “female”. ^∗^*p* < 0.05; ^∗∗^*p* < 0.01; ^∗∗∗^*p* < 0.001.*

**FIGURE 1:**
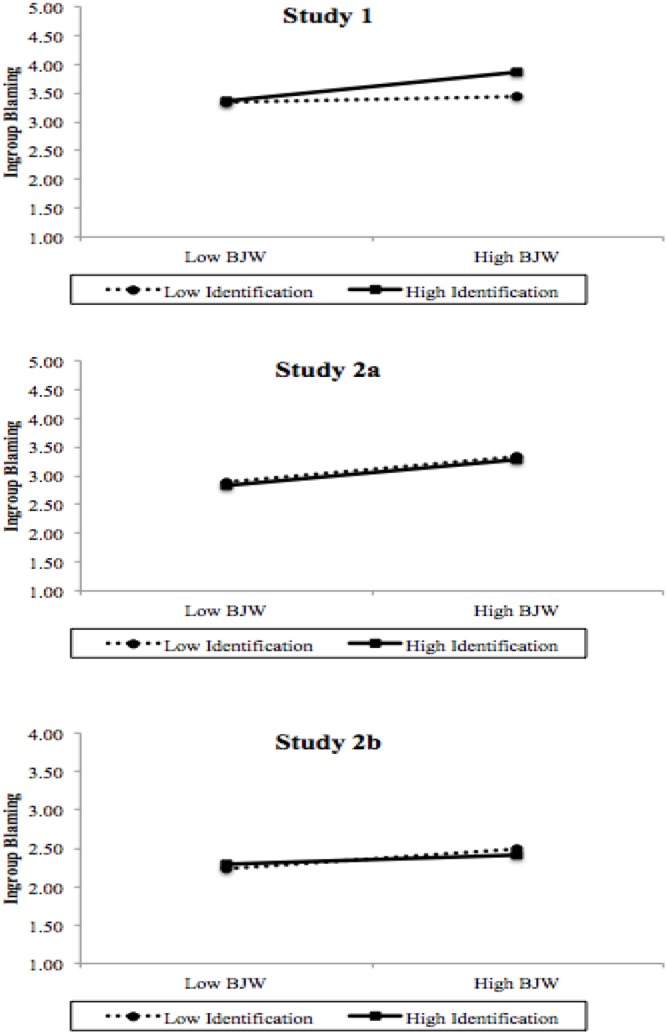
Ingroup blaming as a function of national identification and belief in a just world in each study.

In accordance with our hypothesis, simple slope analyses showed that for Portuguese citizens higher in national identification (i.e., 1 SD above the mean), BJW was positively associated with ingroup blame, *b* = 0.32, *t*(769) = 4.00, *p* = 0.001 (**Figure 1**). Also as we predicted, for Portuguese citizens lower in national identification (i.e., 1 SD below the mean), BJW was not significantly associated with ingroup blame, *b* = 0.06, *t*(769) = 0.74, *p* = 0.461.

The pattern of results we found is the first evidence for the key role played by national identification in understanding the association between BJW and secondary victimization of an ingroup as a whole, especially in a social environment in which the national ingroup is under ongoing suffering imposed by an external outgroup. According to our rationale, this occurred because the more identified participants are, the more they are sensitive to social and psychological consequences of the economic austerity measures, which may have threatened their BJW. Indeed, a bad thing was happening with good people, i.e., with their own beloved and valued ingroup.

Our rationale also assumes that the social environment plays a key role in victimization. Specifically, if the impact of national identification depends on the social environment where the victimization occurs, we can predict that a change in the social context that led to victimization should also impact the association between BJW and ingroup victimization of more national identified individuals. Specifically, if the social environment is less threatening to the ingroup, the need to restore justice should not be so prominent among the most identified individuals, which may mitigate the role of identification in the relationship between BJW and victimization. This possibility will be tested in Study 2.

## Study 2

This study aimed to analyze the moderating role of national identification on the relationship between BJW and ingroup blame in a social context where externally imposed austerity measures were not present anymore. Study 2 was conducted in 2017 when the Troika intervention program had already finished and the Portuguese social environment was regaining some enthusiasm due to returning economic growth. Therefore, the study was conducted in a social context of less victimization compared to that of Study 1. Because of the decreased victimization, it is possible that threat to ingroup had been removed and therefore national identification no longer played a role in the relationship between BJW and ingroup blame. Thus, we predict that national identification should not moderate the BJW effect.

Moreover, Study 2 allows us to overcome some important limitation of the first study. The results we obtained in Study 1 were based on a measure of the dependent variable accessed with only one item, which weakens the accuracy of the estimated parameters and limits the power of inference on the studied phenomenon. The current study addresses this aspect by considering more items to measure ingroup blame, as well as by using different forms of accessing it. The study also tested the proposed hypothesis by taking into account the role played by relevant controlling variables in cross-sectional representative surveys.

### Materials and Methods

#### Participants and Procedure

We used the national Portuguese database from the European Social Survey (ESS) Round 8 (2017). The sample is composed of 1270 individuals who are representative of the Portuguese population. Of them, 1140 indicated they were born in Portugal, have Portuguese nationality, and are over 18 years of age, and so we considered them eligible for the current study. Because we had the possibility to carry out the study with a large and diverse sample, it was possible to extend the test of our hypothesis by using two different versions of the ingroup victimization measure, which allowed us to increase the scope of generality of the proposed effects. Therefore, when the Portuguese specific items of the ESS8 were applied, half of respondents answered a version of victimization items (Subsample A); while the other half responded to a different set of victimization items (Subsample B).

Subsample A is composed of 551 participants aged between 18 and 90 years old (*M* = 53.00, *SD* = 17.85), being 224 male and 327 female (years of schooling, *M* = 10.00, *SD* = 5.38). Subsample B is formed by 589 participants (aged between 18 and 93 years, *M* = 53.60, *SD* = 17.88; 254 male and 335 female; years of schooling, *M* = 9.80, *SD* = 5.41). The participants were randomly allocated either in Subsample A or in Subsample B at the moment they were answering the ingroup victimization items. The two different scales of ingroup blame are presented below.

Participants’ consent was obtained prior to the beginning of the study in accordance with the Declaration of Helsinki and the protocol and questionnaire were approved by the ESS Research Ethics Committee. The data and further information about documentation and data collection can be found at http://www.europeansocialsurvey.org and at http://asp.ics.ul.pt (for the Portuguese Specific Module).

### Measures

#### Belief in a Just World

We asked the participants to answer the same three-items we used in Study 1 to measure BJW (1 = totally agree to 5 = totally disagree). The scores were recoded and averaged so that higher values indicate stronger endorsement of the BJW (Subsample A α = 0.51; Subsample B α = 0.55).

#### Ingroup Identification

We also used the same two items of Study 1 for measuring national identification (Subsample A α = 0.60; Subsample B α = 0.62). We computed a global identification score by averaging across items that vary from 1 (less identification) to 4 (more identification).

#### Ingroup Blaming

As we indicated above, participants were randomly located into two subsamples, according to the set of items we used to measure ingroup blaming. The participants in Subsample A indicated the extent to which they agreed with the following two items by using a five-point answer scale (from 1 = totally agree to 5 = totally disagree): “Regarding the impact of the measures imposed by the Troika in Portugal, Portuguese people are partly to blame for the suffering they went through” and “Regarding the impact of the measures imposed by the Troika in Portugal, Portuguese people are partly responsible for the suffering they went through”. We recoded the answers so that higher scores indicate stronger ingroup blaming. The participants in Subsample B used a four-point answer scale (from 1 = totally agree to 4 = totally disagree) to indicate their agreement with the following two items: “Regarding the impact of the measures imposed by the Troika in Portugal, how much do you think the Portuguese people are to blame for the suffering they went through?”; “Regarding the impact of the measures imposed by the Troika in Portugal, how much do you think the Portuguese people are responsible for the suffering they went through?”. We also recoded the answers so that higher scores indicate stronger ingroup blaming. In both versions, each set of items showed have strong internal consistence: Subsample A (α = 0.85); Subsample B (α = 0.81).

#### Controlling Variables

Besides some relevant participants’ sociodemographic characteristics, we also included three controlling variables: Religiousness (“How often attend religious services apart from special occasions”, coded from 1 = never to 7 = every day); subjective income (“Feeling about present household’s income,” coded from 1 = It is very difficult to live with the present income to 4 = The present household income allows a comfortable life); left–right political positioning, varying from 0 (left) to 10 (right).

#### European Identification

“How close to or identified do you feel with Europe?,” coded from 1 = not at all identified to 4 = very much identified.

### Results and Discussion

#### Preliminary Analysis

**Table 3** shows descriptive statistics and bivariate correlations between all variables. The mean scores of our key variables are substantially different from those we obtained in Study 1, which denote a less threatening social environment. For example, in both samples of the current study, the national and European Identifications were higher than in Study 1 (*t*_s_ > 5.10, *p*_s_ < 0.001; *d*_s_ > 0.27). Similarly, BJW in both Study 2 Subsamples was stronger than in Study 1: *t* = 5.10, *p* < 0.001, *d* = 0.28 and *t* = 5.60, *p* < 0.001, *d* = 0.27, respectively. Importantly, ingroup blaming was significantly lower in Subsample A of Study 2 than in Study 1, *t* = -6.29, *p* < 0.001, *d* = -0.35 (comparison with Subsample B is not adequate because we used a different response scale).^1^

**Table 3:** Correlations and descriptive statistics in Study 2 (Subsample A, *N* = 551; Subsample B, *N* = 589).

	Subsample A		Subsample B		Correlations									
	
Scale	*M*	*SD*	*M*	*SD*	1	2	3	4	5	6	7	8	9	10
(1) Sex	–	–	–	–	–	0.03	–0.05	0.23***	–0.13**	–0.10	0.03	0.05	–0.05	0.04
(2) Age	53.00	17.85	53.65	17.88	0.00	–	–0.54***	0.32**	–0.24***	0.04	0.02	0.23***	–0.10*	–0.09
(3) Year of schooling	10.0	5.38	9.80	5.41	–0.04	–0.54***	–	–0.28***	0.47***	0.08	0.00	–0.21***	–0.07	0.06
(4) Religiousness	2.95	1.71	3.01	1.77	0.19***	0.26***	–0.29***	–	–0.18***	–0.03	0.25***	0.15***	0.08	0.02
(5) Social status	2.82	0.87	2.77	0.89	–0.16***	–0.25***	0.46***	–0.12**	–	0.05	0.06	–0.11**	–0.02	0.07
(6) European Ident.	3.26	1.71	3.31	1.80	–0.05	0.02	0.13**	–0.02	0.11*	–	0.13**	0.25***	0.08	0.12**
(7) Left–right	4.72	2.50	4.58	2.37	0.05	–0.02	0.00	0.19***	0.05	0.07	–	0.02	0.05	0.14**
(8) National Ident.	3.62	0.53	3.65	0.53	0.06	0.19***	–0.18***	0.12**	–0.04	0.22***	–0.05	–	0.04	–0.01
(9) Belief in a just world	2.76	0.73	2.78	0.72	0.02	–0.09*	–0.04	0.05	–0.01	0.06	0.07	0.06	–	0.22***
(10) Ingroup-blame	3.08	1.06	2.34	0.90	–0.07	–0.14**	0.13**	–0.01	0.08	0.10*	0.09*	–0.01	0.12**	–

*Correlation above diagonal were obtained in Subsample A; correlation below the diagonal are in Subsample B. For all variables, higher values indicating stronger endorsement of the construct. For sex, 0 indicates “male” and 1 “female.” ^∗^*p* < 0.05; ^∗∗^*p* < 0.01; ^∗∗∗^*p* < 0.001.*

Regarding the correlations, in each subsample, BJW correlated positively with ingroup blame. The correlation was substantially stronger in Subsample A. In this sample, only BJW and European identification are associated with ingroup blame. In Subsample B, besides the association with BJW, ingroup blame was positively correlated with participants’ years of schooling, subjective income, and identification with Europe, and negatively with age. National identification did not correlate with ingroup blame in any sample, which points in the direction of our hypothesis, according to which ingroup identification plays a less important role in a social context where the victimization is little salient. Despite these preliminary results indicating weak associations between ingroup blame and the controlling variables, we included them in the regression analysis when testing our hypotheses.

#### Main Analysis

**Table 2** presents the estimated parameters for the regression analysis we used to test our prediction in each subsample. The results indicate very consistent results across samples. The more the participants’ BJW, the more they blamed Portuguese people for the Troika intervention. Importantly, this main effect of BJW was not moderated by national identification. This means that the association between BJW and ingroup blame occurred not only over and above individuals’ ingroup identification, but it is equally positive both in more and less identified participants. Moreover, the BJW remained a significant predictor, even taking identification with Europe into account, which was a controlling variable associated with more ingroup blame.

The pattern of results we found in the current study is in accordance with our prediction that the association between BJW and secondary victimization is not moderated by individuals’ national identification in a social environment where ingroup suffering is not salient, as was the case when the study was conducted.^2^ This was at the time the Troika intervention was completed and the country’s socio-economic situation started to get better.

In sum, the results are in accordance with our prediction that BJW is positively associated with ingroup blaming for its own suffering, and that this association is moderated by national identification and by the environmental social context where the secondary victimization occurs. That is, the moderating effect of national identification should, in fact, occur in Study 1, but not in Study 2. In order to carry out a more rigorous test of this hypothesis, we conducted a new analysis in which we assembled the databases of the two studies by focusing on the measures that fully matched between the two time points. We then estimated a regression model taking ingroup blame as the dependent variable, the year when data were collected (-0.5 = 2014; 0.5 = 2017), BJW, national identification (both mean centered), and interaction terms as predictors. Results showed a reliable main effect of the year of study, confirming that ingroup blaming was lower in 2017 than in 2014 (*b* = -0.48, *SE* = 0.07, *t* = -7.34, *p* < 0.001). As expected, we found a reliable main effect of BJW (*b* = 0.27, *SE* = 0.04, *t* = 6.31, *p* < 0.001) and a marginal effect of national identification (*b* = 0.11, *SE* = 0.06, *t* = 1.89, *p* = 0.06). Importantly, we obtained a three-way interaction between the year of study, BJW, and national identification (*b* = -0.29, *SE* = 0.15, *t* = -1.92, *p =* 0.056). As we predicted and verified before in each study, the decomposition of this interaction indicated a reliable two way interaction between BJW and national identification in 2014 (*b* = 0.24, *SE* = 0.09, *t* = 2.64, *p* < 0.01), but not in 2017 (*b* = -0.05, *SE* = 0.12, *t* = -0.40, *p* = 0.69). The pattern of interaction follows those already depicted in **Figure 1**. These results represent strong evidence for our hypothesis that the moderating role played by national identification in the relationship between BJW and ingroup blame depends on the social environment where the victimization occurs.^3^

## General Discussion

This study addressed, for the first time, the relation between national identity and BJW on ingroup blame for externally imposed economic austerity measures by the Troika, that lead to suffering of the population and was perceived as a victimization imposed by an outgroup. Using probabilistic representative samples of Portuguese citizens, we found a consistent pattern of results. Across the two studies, the more participants endorse BJW, the more they blame the ingroup as a whole for their suffering. Importantly, in a social environment characterized by ongoing suffering caused by an outgroup (the Troika) intervention (Study 1), the association of BJW with ingroup blame was moderated by national identification. This result is in accordance with our prediction that BJW is positively associated with ingroup blame only for highly identified Portuguese participants in a social context of victimization by an outgroup. Importantly, 3 years later, when the victimization was less prominent, national identification did not play a role in ingroup blaming (Study 2). In sum, the results were in accordance with our proposal that, in terms of intergroup relations, the moderating role of ingroup identification in the relationship between BJW and secondary victimization depends on the existence of a threatening social context where the ingroup is victimized.

### Theoretical Implications

Results we found provide new insight into the role played by national identification on the relationship between BJW and secondary victimization, establishing a new frontier for understanding the conditions under which ingroup identification favors the relationship between BJW and ingroup blaming.

From a theoretical point of view, the results support the generalizability of the relation between the degree of endorsement of BJW and social identification in the reaction to ingroup victims. Either the ingroup victim is an isolated victim and there is no relation between the nature of the victim’s group and the victimization that happened (Correia et al., 2012); or there is an intrinsic relation between being an ingroup member and being a victim (Correia et al., 2015); or, as in the present study, when people are victimized just because they belong to a given group, the same result was found.

It is also important to stress that these effects have already been obtained with different forms of secondary victimization: victim derogation and psychological distancing from the victims and legitimization of the victimization (Correia et al., 2012, 2015). The present research advances prior research on the psychological effects of victimization, since the moderating role played by ingroup identification occurs when the group as a whole is in continuous suffering (Study 1), but not when the act of victimization by the outgroup was removed (Study 2).

Beyond advancing the literature on secondary victimization, the present paper also contributes to the understanding of the social consequences of intervention imposed by a powerful outgroup, and therefore studies the phenomenon at an intergroup relations level of analysis (Doise, 1986). Our results suggest that the blaming of the ingroup is a way to deal with BJW threat and, consequently, to maintain confidence in the future. Indeed, to believe that the misfortunes occurring with the ingroup are not random and are, to some extent, deserved as a consequence of its own “misbehavior,” may be psychologically and socially functional. In other words, it can contribute to maintaining the fundamental illusion that the events occurring to us are predictable, stable, and controllable (Lerner, 1980).

This possibility is in line with theorizing and research about the socio-psychological consequences of the legitimation of social inequality [see Costa-Lopes et al. (2013) for a review]. From a social identity perspective (Tajfel and Turner, 1979), individuals are motivated to value their ingroup by differentiating it from outgroups in order to maintain positive self-esteem. Individuals who are members of minority groups have their self-esteem threatened when their ingroup is under victimization imposed by a high status outgroup. One possible way to maintain self-esteem is by perceiving victimization as illegitimate and ingroup boundaries as impermeable, which motivates them to engage in collective actions. For instance, when members of minority groups can attribute their misfortune to prejudice, they increase identification with the minority group, which leads to enhanced well-being (e.g., Branscombe et al., 1999); especially those who already have higher ingroup identification (e.g., Bourguignon et al., 2006). Furthermore, highly identified members may deviate from the ingroup members internalization of the disadvantage, willing to improve the ingroup situation (Packer, 2008; Jiménez-Moya et al., 2017).

A different reaction can occur when individuals view victimization as legitimate and group boundaries as permeable. In this case, it motivates them to disidentify with the ingroup, which leads to the outgroup favoritism effect that can have pervasive harmful consequences for the individuals’ own ingroup (Dasgupta, 2004).

The current research shows that individuals can follow a third way in solving the ingroup-victimization problem: they can actively tend to legitimize their situation through ingroup blaming, when their ingroup identity is under threat. Indeed, the self-protective role of national identity motivating a coping process when facing a threat of injustice by an outgroup perpetrator has never been studied, and this paper constitutes a first step in that direction. That possibility is in accordance with the system justification theory that predicts a justice motive to legitimize the existing social order whose function is to reduce dissonance, especially in disadvantaged groups (Jost and Hunyady, 2002). Accordingly, the results of the present research not only extend previous research within the framework of BJW literature but also contribute to illuminate the functionality of BJW in legitimizing the suffering observed in the social system as a whole. It is also in line with recent research that showed at an interpersonal level, that random and uncontrollable bad outcomes increase beliefs about deserving bad outcomes (Callan et al., 2014). Our research contributes to this literature by showing that legitimization also depends on the existence of threats resulting from the macro social environment.

### Limitations and Further Directions

The fact that the constructs were assessed with few items represents a weakness. However, in Study 2 we found the same results with two different measures of ingroup blame. Nevertheless, the results obtained are according to the theoretical predictions, which lessens this issue’s potential impact. Moreover, the size and representativeness of the present study sample allows the hypotheses to be tested on individuals who are very diverse in terms of age, gender, economic status, etc.

We must also not forget that the correlational design of this study limits the nature of the conclusions that can be drawn about the causal and sequential relations among BJW, national identification, and ingroup blame. Future studies should experimentally manipulate the motivation to reestablish the BJW (e.g., Hafer, 2000; Correia and Vala, 2003) and the strength of national identification, to check their joint impact on ingroup blame. Future studies should also include measures related with the perception of efficacy to change the ingroup situation or of support of collective action, so that it is possible to compare high and low identifiers on their perceived efficacy to change the ingroup disadvantaged situation (Jiménez-Moya et al., 2017).

The reliability of BJW in Study 2 was low. The measures of BJW have been mostly used with adult participants with a medium to a high level of education. In our representative sample we have participants with a comparatively lower education level than in most samples. Furthermore, we only have a three-item scale. All these reasons may explain the lower reliability of the BJW scale. Even so, the findings were according to the theoretical predictions.

Methodologically, the main strength of this paper is the analysis of the same population over time, where it is possible to study the impact of threats resulting from the macro social environment on the relation between BJW, national identity, and ingroup blame.

Future studies could also measure the impact of ingroup blame on well-being. In fact, if ingroup blame is used to reduce threat to BJW, it is expected that its use can have positive consequences for the well-being of the individuals. On the downside, it also legitimizes the status quo and contributes to its passive acceptance, and therefore to social injustice.

## Author Contributions

IC, CP, and JV contributed to the conceptualization and design of the research and revised the paper. IC and CP conducted the data analysis.

## Conflict of Interest Statement

The authors declare that the research was conducted in the absence of any commercial or financial relationships that could be construed as a potential conflict of interest.

## Footnotes

1 In Study 1 we had only an item for measure ingroup blaming, while in Study 2 it was possible to measure this construct by using two items. So, we also compared ingroup blaming means between Study 1 and 2 by using only the common item across the two studies. Results demonstrate that, effectively, ingroup blaming was significantly lower in Study 2 (*M* = 3.02; *SD* = 1.16) than in Study 1 (*M* = 3.47; *SD* = 1.15), *t* = -6.39, *p* < 0.001, *d* = -0.4. Moreover, ingroup blaming does not reach the midpoint of the scale in Study 2 (*t* = 0.59, ns, *d* = 0.05), while it was significantly above this midpoint in Study 1 (*t* = 10.88, *p* < 0.001, *d* = 0.73). Additionally, the reduction of ingroup blaming from Study 1 to Study 2 is more evident when we take into account the percentage of participants who agreed with the ingroup blaming indicator. While in Study 1, about 60% of them scored above the midpoint of the scale, in Study 2 this percentage decreases to only 46%.

2 Because in Study 1 we had not an indicator of left–right political placement, the non-significant interaction in Study 2 could be due to control for this variable. In order to address this possibility we re-estimate the regression model without left–right as a controlling variable. Results showed very similar effect to those presented on **Table 2**. In fact, the interaction effect was not significant in Subsample A (*b* = 0.02, *SE* = 0.11, *t* = 0.18, *p* = 0.86), nor in Subsample B (*b* = -0.10, *SE* = 0.11, *t* = -0.97, *p* = 0.33). Thus, the non-moderating effect of national identification in Study 2 cannot be explained by controlling for individuals’ left–right political placement.

3 Moreover, we estimated a regression model after having standardized ingroup blame in each sample, since we have used different metrics for this measure across samples (Study 1, Study 2A, and Study 2B). Results again showed a reliable main effect of BJW (*b* = 0.23, *SE* = 0.03, *t* = 7.75, *p* < 0.001) and confirmed the three-way interaction between year of study, BJW, and national identification (*b* = -0.26, *SE* = 0.11, *t* = -2.37, *p* = 0.018). The decomposition of this interaction also confirmed the significant two-way interaction between BJW and national identification in 2014 (*b* = 0.21, *SE* = 0.08, *t* = 2.64, *p* < 0.01), which was not significant in 2017 (*b* = -0.06, *SE* = 0.08, *t* = -0.70, *p* = 0.48).

## References

[B1] Aguiar P. Vala J. Correia I. Pereira C. (2008). Justice in our world and in other’s world: belief in a just world and reactions to victims. *Soc. Justice Res.* 21 50–68. 10.1007/s11211-007-0059-3

[B2] Aiken L. S. West S. G. (1991). *Multiple Regression: Testing and Interpreting Interactions.* Newbury Park, CA: Sage.

[B3] Bierhoff H. W. Klein R. Kramp P. (1991). Evidence for the altruistic personality from data on accident research. *J. Pers.* 59 263–280. 10.1111/j.1467-6494.1991.tb00776.x

[B4] Bourguignon D. Seron E. Yzerbyt V. Herman G. (2006). Perceived group and personal discrimination: differential effects on personal self-esteem. *Eur. J. Soc. Psychol.* 36 773–789. 10.1002/ejsp.326

[B5] Branscombe N. R. Schmitt M. T. Harvey R. D. (1999). Perceiving pervasive discrimination among African Americans: implications for group identification and well-being. *J. Pers. Soc. Psychol.* 77 135–149. 10.1037/0022-3514.77.1.135

[B6] Callan M. J. Kay A. C. Dawtry R. J. (2014). Making sense of misfortune: deservingness, self-esteem, and patterns of self-defeat. *J. Pers. Soc. Psychol.* 107 142–162. 10.1037/a0036640 24956317 PMC4076324

[B7] Choma B. L. Hafer C. L. Crosby F. Foster M. D. (2012). Perceptions of personal sex discrimination: the role of belief in a just world and discrimination against one’s group. *J. Soc. Psychol.* 152 568–585. 10.1080/00224545.2012.667459 22930997

[B8] Christandl F. (2013). The belief in a just world as a personal resource in the context of inflation and financial crises. *Appl. Psychol.* 62 486–518. 10.1111/j.1464-0597.2012.00489.x

[B9] Correia I. Alves H. Morais R. Ramos M. (2015). The legitimation of wife abuse among women: the impact of belief in a just world and gender identification. *Pers. Individ. Dif.* 76 7–12. 10.1016/j.paid.2014.11.041

[B10] Correia I. Alves H. Sutton R. Ramos M. Gouveia-Pereira M. Vala J. (2012). When do people derogate or psychologically distance themselves from victims? Belief in a just world and ingroup identification. *Pers. Individ. Dif.* 53 747–752. 10.1016/j.paid.2012.05.032

[B11] Correia I. Vala J. (2003). When will a victim be secondarily victimized? The effect of observer’s belief in a just world, victim’s innocence and persistence of suffering. *Soc. Justice Res.* 16 379–400. 10.1023/A:1026313716185

[B12] Correia I. Vala J. Aguiar P. (2007). Victim’s innocence, social categorization and the threat to the belief in a just world. *J. Exp. Soc. Psychol.* 43 31–38. 10.1016/j.jesp.2005.12.010

[B13] Correia I. F. (2003). *Concertos e Desconcertos na Procura de um Mundo Concertado: Crença no Mundo Justo, Inocência da Vítima e Vitimização Secundária [Concerts and Disconcerts in the Search for a Concerted World: Belief in a Just World, Innocence of the Victim and Secondary Victimization].* Lisboa: FCG/FCT

[B14] Costa-Lopes R. Dovidio J. F. Pereira C. R. Jost J. T. (2013). Social psychological perspectives on the legitimation of social inequality: past, present and future. *Eur. J. Soc. Psychol.* 43 229–237. 10.1002/ejsp.19663

[B15] Dalbert C. (2001). *The Justice Motive as a Personal Resource: Dealing with Challenges and Critical Life Events.* New York, NY: Plenum.

[B16] Dalbert C. Montada L. Schmitt M. (1987). Glaube an die gerechte welt als motiv: validierung zweier skalen [Belief in a just world as a motive: validation of two scales]. *Psychol. Beitrage* 29 596–615.

[B17] Dasgupta N. (2004). Implicit ingroup favoritism, outgroup favoritism, and their behavioral manifestations. *Soc. Justice Res.* 17 143–169. 10.1023/B:SORE.0000027407.70241.15

[B18] Doise W. (1986). *Levels of Explanation in Social Psychology.* Cambridge: Cambridge University Press.

[B19] Greenglass E. Antonides G. Christandl F. Foster G. Katter J. K. Q. Kaufman B. E. (2014). The financial crisis and its effects: perspectives from economics and psychology. *J. Behav. Exp. Econ.* 50 10–12. 10.1016/j.socec.2014.01.004

[B20] Hafer C. L. (2000). Do innocent victims threaten the belief in a just world? Evidence from a modified stroop task. *J. Pers. Soc. Psychol.* 79 165–173. 10.1037/0022-3514.79.2.165 10948971

[B21] Hafer C. L. Bègue L. (2005). Experimental research on just-world theory: problems, developments, and future challenges. *Psychol. Bull.* 131 128–167. 10.1037/0033-2909.131.1.128 15631556

[B22] Hafer C. L. Olson J. M. (1989). Beliefs in a just world and reactions to personal deprivation. *J. Pers.* 57 799–823. 10.1111/j.1467-6494.1989.tb00495.x

[B23] INE (2017). *Portugal 2016.* Avaialable at: https://www.ine.pt/xportal/xmain?xpid=INE&xpgid=ine_publicacoes&PUBLICACOESpub_boui=311362272&PUBLICACOESmodo=2

[B24] Jiménez-Moya G. Rodríguez-Bailón R. Spears R. Lemus S. (2017). Collective resistance despite complicity: high identifiers rise above the legitimization of disadvantage by the in-group. *Br. J. Soc. Psychol.* 56 103–124. 10.1111/bjso.12182 28097672

[B25] Jost J. T. Banaji M. R. (1994). The role of stereotyping in system-justification and the production of false consciousness. *Br. J. Soc. Psychol.* 33 1–27. 10.1111/j.20448309.1994.tb01008.x

[B26] Jost J. T. Hunyady O. (2002). The psychology of system justification and the palliative function of ideology. *Eur. Rev. Soc. Psychol.* 13 111–153. 10.1080/10463280240000046

[B27] Lerner M. J. (1980). *The Belief in a Just World: A Fundamental Delusion.* New York, NY: Plenum Press.

[B28] Lerner M. J. Miller D. T. (1978). Just world research and the attribution process: looking back and ahead. *Psychol. Bull.* 85 1030–1051. 10.1037/0033-2909.85.5.1030

[B29] Lerner M. J. Simmons C. H. (1966). Observer’s reaction to the “innocent victim”: Compassion or rejection? *J. Pers. Soc. Psychol.* 4 203–210. 10.1037/h0023562 5969146

[B30] McCoy S. K. Wellman J. D. Cosley B. Saslow L. Epel E. (2013). Is the belief in meritocracy palliative for members of low status groups? Evidence for a benefit for self-esteem and physical health via perceived control. *Eur. J. Soc. Psychol.* 43 307–318. 10.1002/ejsp.1959 24039310 PMC3769703

[B31] Novak D. W. Lerner M. J. (1968). Rejection as a consequence of perceived similarity. *J. Pers. Soc. Psychol.* 9 147–152. 10.1037/h0025850 5662736

[B32] Opotow S. (1995). “Drawing the line: Social categorization, moral exclusion, and the scope of justice,” in *Conflict, Cooperation, and Justice* eds Bunker B. B. Rubin J. Z. (San Francisco, CA: Jossey-Bass) 347–369.

[B33] Packer D. J. (2008). On being both with us and against us: a normative conflict model of dissent in social groups. *Pers. Soc. Psychol. Rev.* 12 50–72. 10.1177/1088868307309606 18453472

[B34] Papastamou S. Valentim J. Mari S. Marchand P. (2018). Socio-cognitive elaborations and reactions to economic crisis: insights from social psychology. *Int. Rev. Soc. Psychol.* 31:4 10.5334/irsp.170

[B35] Sutton R. M. Douglas K. (2005). Justice for all, or just for me? More support for self-other differences in just world beliefs. *Pers. Individ. Dif.* 9 637–645. 10.1016/j.paid.2005.02.010

[B36] Tajfel H. Turner J. C. (1979). “An integrative theory of intergroup conflict,” in *The Social Psychology of Intergroup Relations* eds Austin W. G. Worchel S. (Monterey, CA: Brooks-Cole).

[B37] Turner J. C. Hogg M. A. Oakes P. J. Reicher S. D. Wetherell M. S. (1987). *Rediscovering the Social Group. A Self-Categorization Theory.* Oxford: Blackwell.

